# Complete genome sequence of the *Medicago* microsymbiont *Ensifer* (*Sinorhizobium*)* medicae* strain WSM419

**DOI:** 10.4056/sigs.43526

**Published:** 2010-01-28

**Authors:** Wayne Reeve, Patrick Chain, Graham O’Hara, Julie Ardley, Kemanthi Nandesena, Lambert Bräu, Ravi Tiwari, Stephanie Malfatti, Hajnalka Kiss, Alla Lapidus, Alex Copeland, Matt Nolan, Miriam Land, Loren Hauser, Yun-Juan Chang, Natalia Ivanova, Konstantinos Mavromatis, Victor Markowitz, Nikos Kyrpides, Margaret Gollagher, Ron Yates, Michael Dilworth, John Howieson

**Affiliations:** 1 Centre for Rhizobium Studies, Murdoch University, Perth, Australia; 2 DOE Joint Genome Institute, Walnut Creek, California, USA; 3 Lawrence Livermore National Laboratory, Livermore, California, USA; 4 Oak Ridge National Laboratory, Oak Ridge, Tennessee, USA; 5 Biological Data Management and Technology Center, Lawrence Berkeley National Laboratory, Berkeley, California, USA; 6 Institute for Sustainability and Technology Policy, Murdoch University, Perth, Australia; 7 Department of Agriculture and Food, South Perth, Australia

**Keywords:** microsymbiont, non-pathogenic, aerobic, Gram-negative rod, root-nodule bacteria, nitrogen fixation, *Alphaproteobacteria*

## Abstract

*Ensifer* (*Sinorhizobium*)* medicae* is an effective nitrogen fixing microsymbiont of a diverse range of annual *Medicago* (medic) species. Strain WSM419 is an aerobic, motile, non-spore forming, Gram-negative rod isolated from a *M. murex* root nodule collected in Sardinia, Italy in 1981. WSM419 was manufactured commercially in Australia as an inoculant for annual medics during 1985 to 1993 due to its nitrogen fixation, saprophytic competence and acid tolerance properties. Here we describe the basic features of this organism, together with the complete genome sequence, and annotation. This is the first report of a complete genome sequence for a microsymbiont of the group of annual medic species adapted to acid soils. We reveal that its genome size is 6,817,576 bp encoding 6,518 protein-coding genes and 81 RNA only encoding genes. The genome contains a chromosome of size 3,781,904 bp and 3 plasmids of size 1,570,951 bp, 1,245,408 bp and 219,313 bp. The smallest plasmid is a feature unique to this medic microsymbiont.

## Introduction

Agricultural systems are nearly always nitrogen deficient, a factor which grossly limits their productivity. In fact, each year some 50 Tg of nitrogen is harvested globally in food crops [3], and must be replaced. External inputs of nitrogen  to agriculture may come from mineral fertilizers, the production of which is heavily dependent on fossil fuels. Alternatively, nitrogen can be obtained from symbiotic nitrogen fixation (SNF) by root nodule bacteria (rhizobia) on nodulated legumes [4]. SNF is therefore considered a key biological process on the planet. The commonly accepted figure for global SNF in agriculture is 50-70 million metric tons annually, worth in excess of U.S. $10 billion [5]. Rhizobia associated with forage legumes contribute a substantial proportion of this fixed nitrogen across 400 million ha [5]. The amount fixed annually by the *Ensifer* (*Sinorhizobium*)-*Medicago* symbiosis is estimated to be worth $250 million.

A particular constraint to the formation of this symbiosis is acidity, due mainly to the acid-sensitive nature of the microsymbionts [6]. In laboratory culture, the medic microsymbionts fail to grow below pH 5.6 and are considered to be the most acid-sensitive of all the commercial root nodule bacteria [7]. Many agricultural regions have moderately acidic soils (typically in the pH range of 4.0 to 6.0) and this has prevented the *Ensifer*-*Medicago* symbiosis reaching its full potential [8]. Consequently, an effort was initiated in the 1980s to discover more acid-tolerant medic microsymbionts from world regions with acidic soils upon which annual medics had evolved. A particular suite of strains isolated from acidic soils on the Italian island of Sardinia proved to be acid soil tolerant [9], an attribute we now know is related to the presence of a unique set of genes required for acid adaptation [10]. Characterization of these acid-tolerant isolates revealed that they belonged to the species *E. medicae* and could be symbiotically distinguished from the related species *E. meliloti* by their unique capacity to fix nitrogen in association with annual acid soil adapted *Medicago* hosts of worldwide agronomic value [11], as well as with the perennial forage legume *M*. *sativa* (alfalfa) [12].

One of the acid-tolerant isolates, *E. medicae* strain WSM419, was isolated in 1981 from a nodule recovered from the roots of an annual medic (*M. murex*) growing south of Tempio in Sardinia. WSM419 is of particular interest because it is saprophytically competent in the acidic, infertile soils of southern Australia [9,13], and it is also a highly effective nitrogen  fixing microsymbiont of a broad range of annual medics of Mediterranean origin [11,12]. These attributes contributed to the commercialization of the strain in Australia as an inoculant for acid soil medics between 1985 and 1993 [14,15]. Here we present a summary classification and a set of features (Table 1) for *E. medicae* strain WSM419, together with the description of a complete genome sequence and annotation.

**Table 1:** Classification and general features of *E. medicae* WSM419 according to the MIGS recommendations [16].

**MIGS ID**	**Property**	**Term**	**Evidence code**
	Current classification	Domain *Bacteria*	TAS [17]
		Phylum *Proteobacteria*	TAS [18]
		Class *Alphaproteobacteria*	TAS [19,20]
		Order *Rhizobiales*	TAS [20,21]
		Family *Rhizobiaceae*	TAS [22,23]
		Genus *Ensifer*	TAS [1,2,24-27]
		Species *Ensifer medicae*	TAS [1,2,11,24-28]
		strain WSM419	
	Gram stain	negative	TAS [29]
	Cell shape	rod	TAS [29]
	Motility	motile	TAS [29]
	Sporulation	non-sporulating	TAS [29]
	Temperature range	mesophile	TAS [29]
	Optimum temperature	28°C	TAS [29]
	Salinity	unknown	
MIGS-22	Oxygen requirement	aerobic	TAS [29]
	Carbon source	galactose, arabinose, glutamate	TAS [9,13]
	Energy source	chemoorganotroph	TAS [9,13]
MIGS-6	Habitat	Soil, root nodule, host	TAS [9]
MIGS-15	Biotic relationship	Free living or symbiotic	TAS [9]
MIGS-14	Pathogenicity	none	TAS [16]
	Biosafety level	1	TAS [30]
	Isolation	*Medicago murex* root nodule	TAS [9]
MIGS-4	Geographic location	Forestry Station 7 km south of Tempio, Sardinia, Italy	TAS [9]
MIGS-5	Nodule collection date	May 1^st^, 1981	TAS [31]
MIGS-4.1 MIGS-4.2	Longitude Latitude	9.101915 40.888925	TAS [31]
MIGS-4.3	Depth	<10 cm	TAS [31]
MIGS-4.4	Altitude	350m	TAS [31]

Evidence codes - IDA: Inferred from Direct Assay (first time in publication); TAS: Traceable Author Statement (i.e., a direct report exists in the literature); NAS: Non-traceable Author Statement (i.e., not directly observed for the living, isolated sample, but based on a generally accepted property for the species, or anecdotal evidence). These evidence codes are from the Gene Ontology project [32]. If the evidence code is IDA, then the property was directly observed for a living isolate by one of the authors or an expert mentioned in the acknowledgements.

## Classification and features

**Figure 1:**
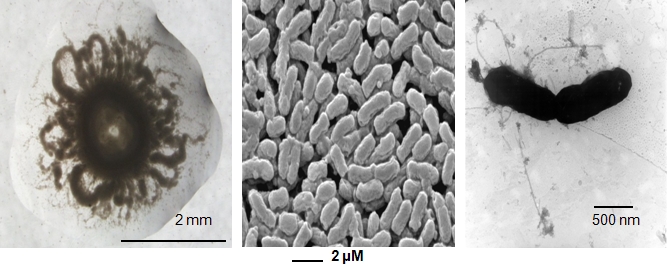
Unique colony morphology (Left) and scanning (Center) and transmission (Right) electron micrographs of *E. medicae* strain WSM419.

*E. medicae* strain WSM419 forms mucoid colonies that may appear as donut shaped (Figure 1, left) on specific media such as YMA [13]. It is a Gram-negative, non-spore-forming rod (Figure 1, center) that has peritrichous flagellae (Figure 1, right).

**Figure 2:**
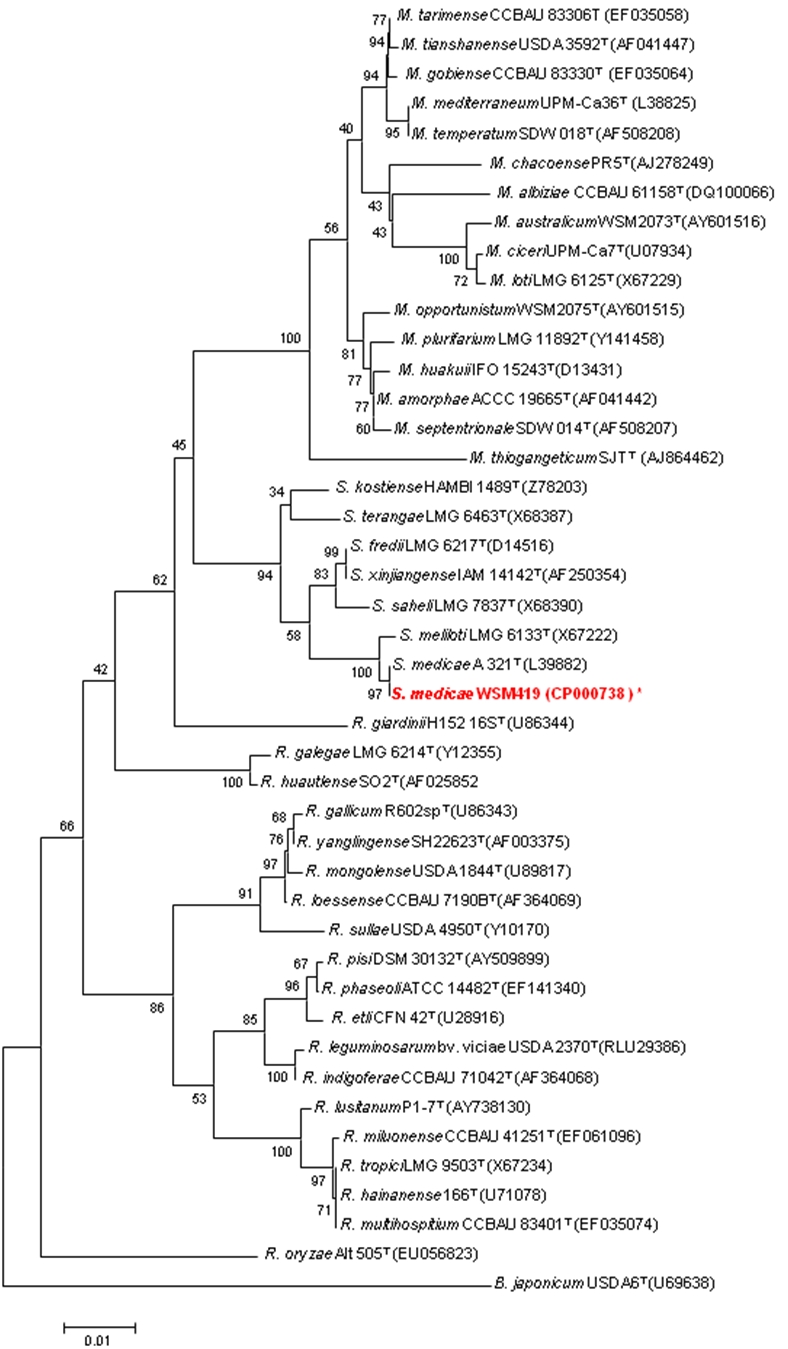
Phylogenetic tree showing the relationships of *E. medicae* strain WSM419 to type strains in the *Rhizobiaceae* based on aligned sequences of the 16S rRNA gene (1,440 bp internal region). All sites were informative and there were no gap-containing sites. Phylogenetic analyses were performed using MEGA, version 3.1 [34]. Kimura two-parameter distances were derived from the aligned sequences [35] and a bootstrap analysis [36] as performed with 500 replicates in order to construct a consensus unrooted tree using the neighbor-joining method [37] for each gene alignment separately. Genera in this tree include *Bradyrhizobium* (B); *Mesorhizobium* (M); *Rhizobium* (R); *Ensifer* (*Sinorhizobium*) (S). Type strains are indicated with a superscript T. Strains with a genome sequencing project registered in GOLD [31] are in bold red print. Published genomes are designated with an asterisk.

In minimal media *E. medicae* WSM419 has a mean generation time of 4.1 h when grown at 28°C [33]. It is a member of the *Rhizobiaceae* family of the class *Alphaproteobacteria* based on phylogenetic analysis. Figure 2 shows the phylogenetic neighborhood of *E. medicae* strain WSM419 inferred from a 16S rRNA based phylogenetic tree. An intragenic fragment of 1,440 bp was chosen since the 16S rRNA gene has not been completely sequenced in many type strains. A comparison of the entire 16S rRNA gene of WSM419 to completely sequenced 16S rRNA genes of other sinorhizoabia revealed 4 and 18 bp mismatches to the reported sequences of *E. meliloti* (Sm1021) and *E. fredii* (YcS2, 15067 and SjzZ4), respectively.

### Symbiotaxonomy

*E. medicae* and *E. meliloti* are traditionally separated on the basis of the effective nodulation (Nod^+^, Fix^+^) by *E. medicae* on *M. polymorpha* [38]. Specific symbiotic characteristics that further distinguish *E. medicae* WSM419 from *E. meliloti* include its ability to nodulate and fix nitrogen effectively with a wide range of annual Mediterranean medics, including *M. polymorpha, M. arabica, M. murex* and *M. sphaerocarpos*. WSM419 is symbiotically competent with these species when grown in acidic soils [39]. In contrast, WSM419 is Fix^-^ with the alkaline soil species of annual medics such as *M. littoralis, M. tornata* and hybrids of *M. littoralis/M. truncatula* [11,40]. WSM419 is also Nod^+^, Fix^+^ with the perennial forage legume *M. sativa* [11,12] but is less effective with this species than are some *E. meliloti* isolates. However, WSM419 is more effective at fixing nitrogen with *M. truncatula* than the previously sequenced *E. meliloti* Sm1021, making it an ideal candidate for inoculation of this model legume [12].

## Genome sequencing and annotation

### Genome project history

*E. medicae* WSM419 was selected for sequencing on the basis of its importance as a symbiotic nitrogen fixing bacterium in agriculture, and its tolerance for acidic soils [9,14].This strain was selected for sequencing as part of the Community Sequencing Program of the Joint Genome Institute (JGI) in 2005. The genome project is deposited in the Genomes OnLine Database [31] and the complete genome sequence in GenBank. A summary of the project information is shown in Table 2.

**Table 2:** Genome sequencing project information of *E. medicae* WSM419.

MIGS ID	Property	Term
MIGS-31	Finishing quality	Finished
MIGS-28	Libraries used	Four Sanger libraries – 3 kb pUC18, 2 kb pTH1522, 8 kb pMCL200 and fosmid pCC1Fos
MIGS-29	Sequencing platforms	ABI3730xl; MegaBACE4500
MIGS-31.2	Sequencing coverage	~13× Sanger
MIGS-30	Assemblers	PHRED/PHRAP/CONSED
MIGS-32	Gene calling method	Critica, Generation and Glimmer
	Genbank ID	CP000738 (Chromosome)^a^ CP000739 (pSMED01 or pSymB)^b^ CP000740 (pSMED02 or pSymA)^c^ CP000741 (pSMED03 or accessory plasmid)^d^
	Genbank Date of Release	June 29, 2007
	GOLD ID	Gc00590^e^
	NCBI project ID	16304
	Database: IMG	640753051f^f^
	Project relevance	Symbiotic nitrogen fixation, agriculture

^a^ http://www.ncbi.nlm.nih.gov/nuccore/150026743

^b^ http://www.ncbi.nlm.nih.gov/nuccore/150030273

^c^ http://www.ncbi.nlm.nih.gov/nuccore/150031715

^d^ http://www.ncbi.nlm.nih.gov/nuccore/150032810

^e^ http://genomesonline.org/GOLD_CARDS/Gc00590.html

^f^ http://img.jgi.doe.gov/cgi-bin/pub/main.cgi?page=taxonDetail&taxon_oid=640753051

### Growth conditions and DNA isolation

*E. medicae* strain WSM419 was grown to mid logarithmic phase in TY medium (a rich medium) [41] on a gyratory shaker at 28°C. DNA was isolated from 60 ml of cells using a CTAB (Cetyl trimethylammonium bromide) bacterial genomic DNA isolation method (http://my.jgi.doe.gov/general/index.html).

### Genome sequencing and assembly

The genome was sequenced using a Sanger platform. All general aspects of library construction and sequencing performed at the JGI can be found at the JGI website (http://www.jgi.doe.gov/). Sequence data statistics from the trace archive for this project are presented in Table 3.

**Table 3:** Production sequence data for the *E. medicae* WSM419 genome project (JGI project 4001622).

**Library**	**Vector**	**Insert size(kb)**	**Reads**	**Mb**	**q20 (Mb)**
BICH	pMCL200	5.9	37,091	36.3	25.7
BICG	pUC18c	2.6	33,520	36.8	26.1
BICI	pCC1Fos	38.8	13,929	13.9	8.9
FAUT	pTH1522	2.1	7,376	6.4	5.4
			91,916	93.4	66.1

All reads were assembled using the phrap assembler. Possible mis-assemblies were corrected and gaps between contigs were closed by custom primer walks from sub-clones or PCR products. Processing of sequence traces and base calling and assessment of data quality and assembly were performed with the PHRED/PHRAP/CONSED package [42-44]. The initial draft assembly was produced from 84,192 high-quality reads and consisted of 30 contigs (each with at least 20 reads per contig). Gaps in the sequence were primarily identified by mate-pair sequences and then closed by primer walking on gap-spanning library clones or genomic DNA amplified PCR products. True physical gaps were closed by combinatorial and multiplex PCR. All repeated sequences were addressed using mate-pair sequences and PCR data. Sequence finishing and polishing added 638 reads. The final assembly of the main chromosome and 3 plasmids from 84,830 reads produced approximately 13-fold coverage across the genome. Assessment of final assembly quality was completed as described previously [45].

### Genome annotation

Automated gene prediction was completed by assessing congruence of gene call results from three independent programs, the Critica [46], Generation, and Glimmer [47] modeling packages, and by comparing the translations to the GenBank nonredundant database using the basic local alignment search tool for proteins (BLASTP). Product description annotations were obtained using searches against the KEGG, InterPro, TIGRFams, PROSITE, and Clusters of Orthologous Groups of protein (COGs) databases. The tRNAScanSE tool [48] was used to find tRNA genes, whereas ribosomal RNAs were found by using BLASTN vs. the 16S and 23S ribosomal RNA databases. Initial comparative analyses of bacterial genomes and gene neighborhoods were completed using the JGI Integrated Microbial Genomes web-based interface (http://img.jgi.doe.gov/cgi-bin/pub/main.cgi). Additional gene prediction analysis and functional annotation was performed within the Integrated Microbial Genomes (IMG-ER) platform (http://img.jgi.doe.gov/er) [49].

### Genome properties

**Figure 3:**
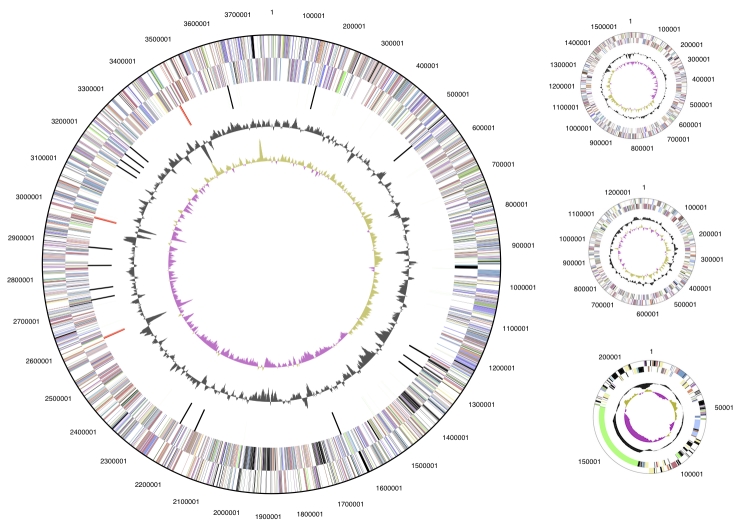
Graphical circular map of the chromosome  and plasmids of *E. medicae* WSM419. From outside to the center: Genes on forward strand (color by COG categories as denoted in the IMG platform), Genes on reverse strand (color by COG categories), RNA genes (tRNAs green, sRNAs red, other RNAs black), GC content, GC skew. The replicons are not drawn to scale.

The genome is 6,817,576 bp long with 61.15% GC content and comprised of four replicons (Table 4); one circular chromosome of size 3,781,904 bp (Figure 3) and three plasmids of size 1,570,951 bp, 1,245,408 bp and 219,313 bp (Figure 4). Of the 6,599 genes predicted, 6,518 were protein-coding genes, and 81 RNA only encoding genes. In addition, 305 pseudogenes were identified. The majority of the genes (70.4%) were assigned a putative function while those remaining were annotated as hypothetical proteins. The distribution of genes into COGs functional categories is presented in Table 5.

**Table 4:** Genome Statistics for *E. medicae* WSM419.

**Attribute**	**Value**	**% of Total**
Genome size (bp)	6,817,576	100.00
DNA coding region (bp)	6,001,805	88.03
DNA G+C content (bp)	4,168,935	61.15
Number of replicons	4	100.00
Extrachromosomal elements	3	75.00
Total genes	6,599	100.00
RNA genes	81	1.23
rRNA operons	3	
Protein-coding genes	6,518	98.77
Pseudo genes	305	4.62
Genes with function prediction	4,646	70.40
Genes in paralog clusters	4,138	62.71
Genes assigned to COGs	4,999	75.75
Genes assigned Pfam domains	5,051	76.54
Genes with signal peptides	2,170	32.88
Genes with transmembrane helices	1,481	22.44
CRISPR repeats	0	

**Table 5:** Number of protein encoding genes of *E. medicae* WSM419 associated with the 21 general COG functional categories.

**Code**	**value**	**% age**	**Description**
J	182	2.79	Translation, ribosomal structure and biogenesis
A	0	0.00	RNA processing and modification
K	501	7.69	Transcription
L	250	3.84	Replication, recombination and repair
B	1	0.02	Chromatin structure and dynamics
D	36	0.55	Cell cycle control, mitosis and meiosis
Y	0	0.00	Nuclear structure
V	56	0.86	Defense mechanisms
T	247	3.79	Signal transduction mechanisms
M	287	4.40	Cell wall/membrane biogenesis
N	66	1.01	Cell motility
Z	0	0.00	Cytoskeleton
W	1	0.02	Extracellular structures
U	106	1.63	Intracellular trafficking and secretion
O	178	2.73	Posttranslational modification, protein turnover, chaperones
C	336	5.15	Energy production and conversion
G	582	8.93	Carbohydrate transport and metabolism
E	622	9.54	Amino acid transport and metabolism
F	109	1.67	Nucleotide transport and metabolism
H	196	3.01	Coenzyme transport and metabolism
I	209	3.21	Lipid transport and metabolism
P	296	4.54	Inorganic ion transport and metabolism
Q	159	2.44	Secondary metabolites biosynthesis, transport and catabolism
R	687	10.54	General function prediction only
S	528	8.10	Function unknown
-	1,519	23.30	Not in COGs
